# Extraction of Flavonoids From Natural Sources Using Modern Techniques

**DOI:** 10.3389/fchem.2020.507887

**Published:** 2020-09-25

**Authors:** Jaísa Oliveira Chaves, Mariana Corrêa de Souza, Laise Capelasso da Silva, Daniel Lachos-Perez, Paulo César Torres-Mayanga, Ana Paula da Fonseca Machado, Tânia Forster-Carneiro, Mercedes Vázquez-Espinosa, Ana Velasco González-de-Peredo, Gerardo Fernández Barbero, Mauricio Ariel Rostagno

**Affiliations:** ^1^Multidisciplinary Laboratory in Food and Health, School of Applied Sciences, University of Campinas, Limeira, Brazil; ^2^Laboratory of Optimization, Design and Advanced Control - Bioenergy Research Program, School of Chemical Engineering, University of Campinas, Campinas, Brazil; ^3^School of Food Engineering, University of Campinas, Campinas, Brazil; ^4^Facultad de Ingeniería, Universidad Nacional Micaela Bastidas de Apurímac, Abancay, Peru; ^5^Department of Analytical Chemistry, Faculty of Sciences, University of Cadiz, Cadiz, Spain

**Keywords:** flavonoids, natural products, extraction, sample preparation, modern techniques

## Abstract

Flavonoids are one of the main groups of polyphenols found in natural products. Traditional flavonoid extraction techniques are being replaced by advanced techniques to reduce energy and solvent consumption, increase efficiency and selectivity, to meet increased market demand and environmental regulations. Advanced technologies, such as microwaves, ultrasound, pressurized liquids, supercritical fluids, and electric fields, are alternatives currently being used. These modern techniques are generally faster, more environmentally friendly, and with higher automation levels compared to conventional extraction techniques. This review will discuss the different methods available for flavonoid extraction from natural sources and the main parameters involved (temperature, solvent, sample quantity, extraction time, among others). Recent trends and their industrial importance are also discussed in detail, providing insight into their potential. Thus, this paper seeks to review the innovations of compound extraction techniques, presenting in each of them their advantages and disadvantages, trying to offer a broader scope in the understanding of flavonoid extraction from different plant matrices.

## Introduction

Flavonoids are a class of natural phenolic compounds synthesized in plants as bioactive secondary metabolites (Nabavi et al., 2018) that are responsible for the characteristics of flavor, color, and pharmacological activities (Scarano et al., 2018). They are potent antioxidants protecting plants from unfavorable environmental conditions (Nabavi et al., 2018). Studies have shown that flavonoids have immunomodulatory, anti-inflammatory (Yahfoufi et al., 2018) and anticancer activities (Abotaleb et al., 2018; Chirumbolo et al., 2018; Rodriguez-Garcia and Sanchez-Quesada, 2019).

**Figure 1:**
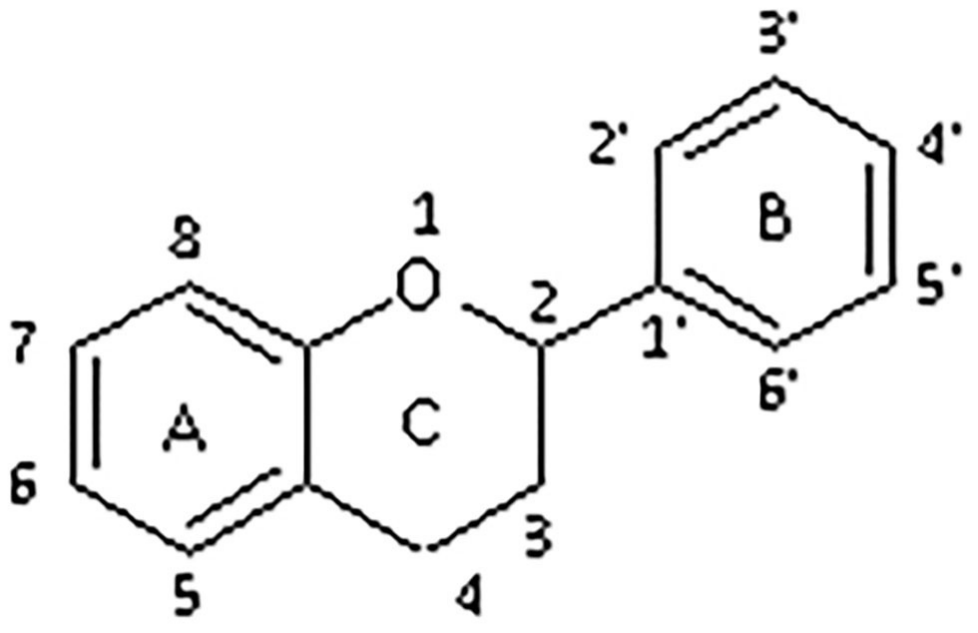
Chemical structure of flavonoid.

**Figure 2:**
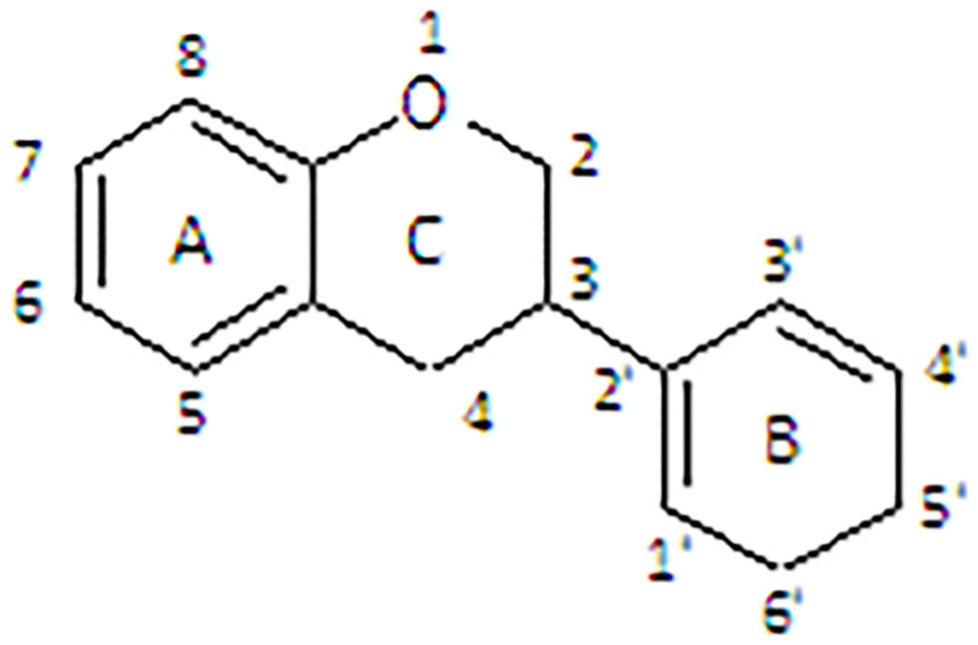
Chemical structure of isoflavonoid.

All flavonoids are based on a fifteen-carbon flavone skeleton C6 (A ring)-C3 (C ring)-C6 (B ring), composed by two benzene rings (A and B) connected by a heterocyclic pyrene ring (C) containing oxygen, as shown in Figure 1. They can be grouped into different classes (flavonols, isoflavones, flavones, chalcones, flavanones, and anthocyanidins) (Table 1), depending on the carbon of the C-ring in which the B-ring is bound and the degree of saturation and oxidation of the C-ring (Panche et al., 2016; Abotaleb et al., 2018; Durazzo et al., 2019). The various classes of flavonoids differ in the oxidation level and substitution pattern of the C ring, while individual compounds within a class differ in the substitution pattern of the A and B rings (Kumar and Pandey, 2013; Panche et al., 2016). A chromane ring (A and C) is attached to a B ring (Figure 1) at C2 in flavonoids or C3 in isoflavonoids (Panche et al., 2016) (Figure 2).

**Table 1:** Classification of flavonoids and their classes according to their skeletal structure.

**Flavonoid group**	**Compound**	**Skeletal structure**	**3′**	**4′**	**5′**	**3**	**5**	**7**
Flavonol	Quercetin	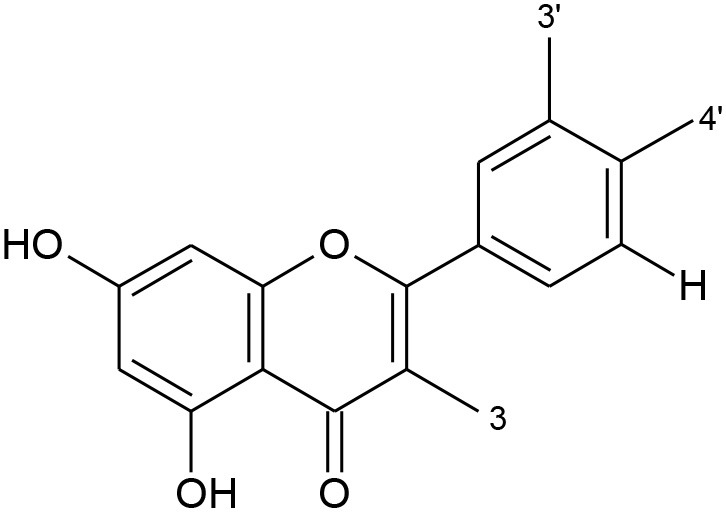	OH	OH	–	OH	–	–
	Isorhamnetin		O-CH_3_	OH	–	OH	–	–
	Kaempferol		H	OH	–	OH	–	–
	Spiraeoside		OH	O-glucose	–	OH	–	–
	Quercitrin		OH	OH	–	O-rhamnose	–	–
	Isoquercitrin		OH	OH	–	O-glucose	–	–
Isoflavone	Genistein	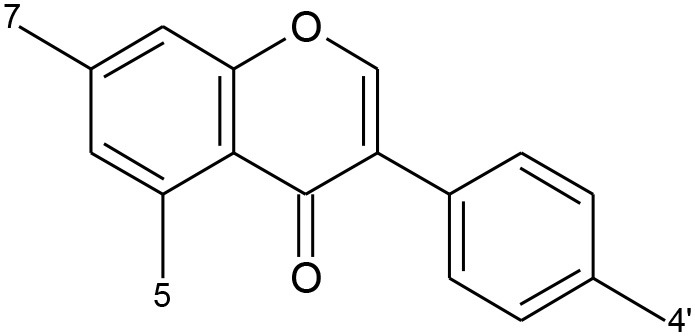	–	OH	–	–	OH	OH
	Genistin		–	OH	–	–	OH	O-glucose
	Daidzein			OH	–	–	–	OH
	Daidzin			OH	–	–	–	O-glucose
	Biochanin A			O-CH_3_	–	–	OH	OH
	Formononetin			O-CH_3_	–	–	–	OH
Flavone	Apigenin	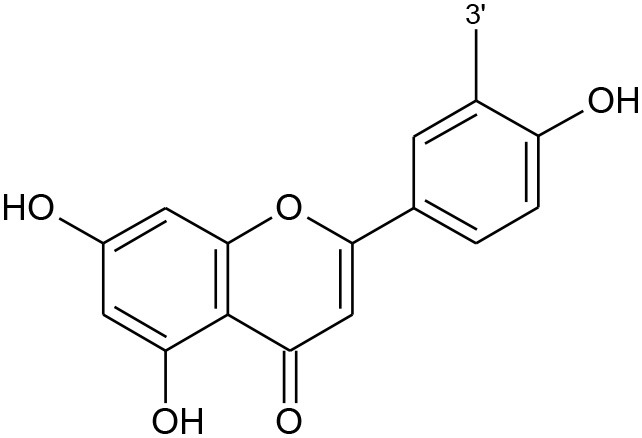	H	–	–	–	–	–
	Luteolin		OH	–	–	–	–	–
Chalcones	Phloretin	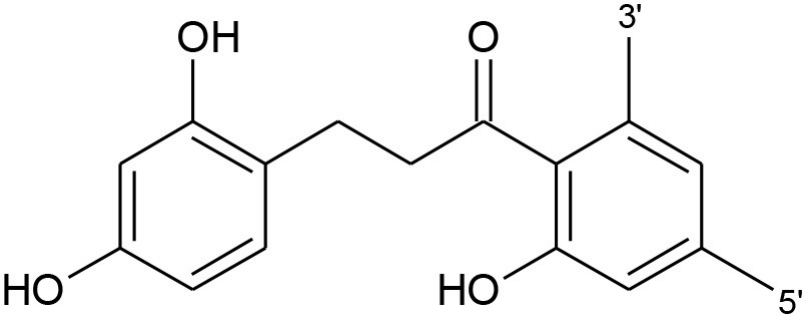	OH	–	OH	–	–	–
	Chalconarigenin	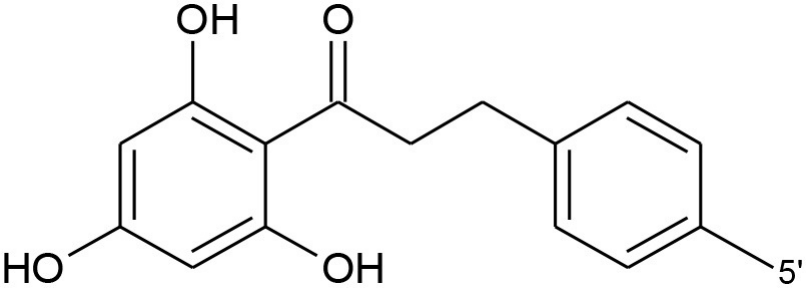	–	–	OH	–	–	–
Flavanone	Narigenin	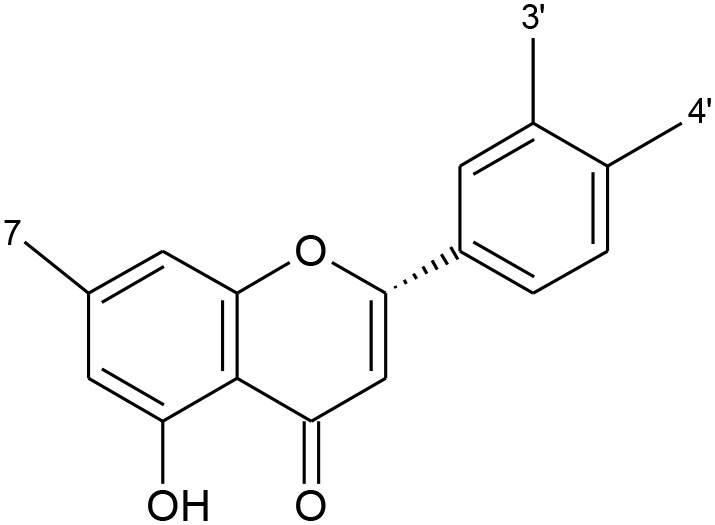	H	OH	–	–	–	OH
	Hesperitin		OH	O-CH_3_	–	–	–	OH
	Narirutin		H	OH	–	–	–	O-rutinoside
	Naringin		H	OH	–	–	–	O-neohesperidose
	Hesperidin		OH	O-CH_3_	–	–	–	O-rutinoside
Anthocyanins	Cyanidin	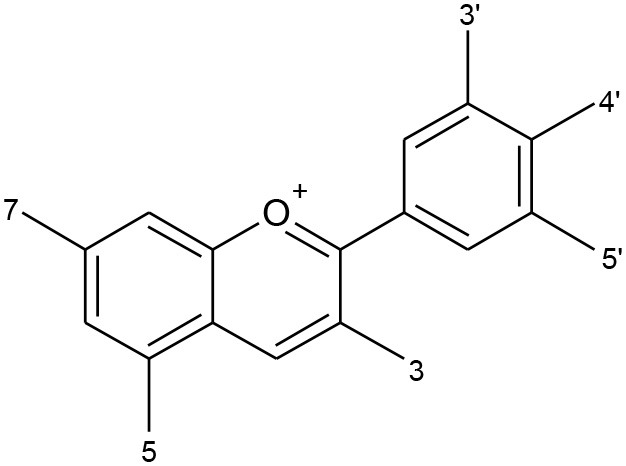	OH	OH	–	OH	OH	OH
	Cyanin		OH	OH	–	O-glucose	OH	OH
	Delphinidin		OH	OH	OH	OH	OH	OH
	Pelargonidin		–	OH	OH	OH	OH	OH

Based on their potential and the importance of foreseen applications, researchers have been extensively studying techniques and conditions specifically for the extraction of flavonoids from natural products and foods either for analytical, preparative, or industrial purposes. Therefore, several techniques for extracting flavonoids to increase the extraction yields of these major bioactive compounds have been implemented. Various techniques have been proposed, including maceration, percolation, hydro-distillation, boiling, reflux, soaking, and soxhlet (Alara et al., 2018a). However, these techniques are characterized by the use of large amounts of organic solvents with high purity, lower extraction yields, low selectivity, long extraction times, thermal degradation of target compounds, associated environmental concerns and costs, compared to other techniques ( et al., 2010; Galanakis, 2012; Farzaneth and Carvalho, 2017).

Advanced techniques and strategies are continually being developed to overcome the limitations of conventional methods for the extraction of these compounds. These techniques include ultrasound-assisted extraction (UAE), supercritical fluid extraction (SFE), microwave-assisted extraction (MAE), solid-phase extraction (SPE), enzyme-assisted extraction, pressurized liquid extraction (PLE or accelerated solvent extraction—ASE), extraction assisted by pulsed electric field (PEF), and a combination of different techniques. These modern techniques are very effective in the removal of flavonoids from the most diverse types of natural matrices. More importantly, they also allow reducing the use of organic solvents and replacing them with alternative “green” solvents while achieving high yields in short extraction times. Therefore, these techniques can be categorized as “green extraction” techniques (Chemat et al., 2012; Khoddami et al., 2013; Galván et al., 2018; Alexandre et al., 2020; Dzah et al., 2020; Pashazadeh et al., 2020; Sarfarazi et al., 2020; Sengar et al., 2020; Tungmunnithum et al., 2020).

There are several reviews available in the literature focusing on one or more extraction techniques, compounds and compound classes and tissue type or plant (Rostagno et al., 2009; Chan et al., 2011; Mustafa and Turner, 2011; Miljevic et al., 2014; Barba et al., 2016; Kala et al., 2016; Roohinejad et al., 2016; Sookjitsumran et al., 2016; Vinatoru et al., 2017; Galván et al., 2018; Dzah et al., 2020).

Due to the complex nature of sample matrix and diverse chemical characteristics of flavonoids, it is consensual among scholars in this field that there is no single and standard method to be used for every material or flavonoids to be extracted at this time.

Thus, this article seeks to revise the innovations of the techniques available, offering a broader scope in the understanding of the extraction of flavonoids from different matrices to be explored by researchers working in this field.

## Advanced Extractions Techniques

### Ultrasound-Assisted Extraction (UAE)

#### Fundamentals

Ultrasound is an intensification technique which is widely used for the extraction of bioactive compounds from natural products with applications in industries such as food and pharmaceuticals (Akbari, 2019). The intensification process is based on the acoustic cavitation phenomena, which consists of the formation of stable or transient gas bubbles by the compression and expansion cycles caused by the passage of ultrasonic waves through the liquid (Leong et al., 2011) and the subsequent rupture, which causes the release of bioactive compounds, and this rupture depends on the extraction conditions (Chen X. J. et al., 2007; Um et al., 2018). As bubbles accumulate energy, they reach a critical point where they implode and release this energy instantaneously, breaking intermolecular interactions between target compounds and the matrix of the sample as well as causing mechanical effects in the extraction medium and the structure of the sample matrix. The mechanical effects include the reduction of particle size and damage of cells, allowing more significant interaction between the solvent and the sample, and consequently, improving mass transfer, which in turn will return higher yields in shorter times (Um et al., 2018). Increased solubilization of the mixture formed in the extraction is also one of the effects of cavitation since the ultrasonic waves allow movement of the liquid from shear forces and turbulence caused by bubbles imploding (Vilkhu et al., 2011; Meullemiestre et al., 2016).

In general, UAE can be explored in more sustainable processes due to the high efficiency associated with its use, allowing lower consumption of solvents and energy. UAE also provides faster extractions, with high reproducibility, rapid return on investment, simplification of manipulation and processing, and higher purity of the final product when compared to conventional extraction methods (Palma et al., 2013; Chemat et al., 2017). UAE can be used in many types of matrices, such as fruits, teas, seeds, vegetables, or flowers, which are sources of many bioactive compounds of different classes (Shirsath et al., 2012; Tabaraki et al., 2012). However, the conditions and the combinations of the variables of the process must be cautiously defined according to the type of sample and compound to be extracted, including factors such as the frequency used and usually between 20 kHz and 100 MHz, ultrasound power, time, temperature, quantity and preparation of the sample, and selection, volume, and concentration of solvent (Rostagno et al., 2010; Da Porto et al., 2013).

#### Parameters Influencing UAE Processes

The extraction of natural products is a complex process, where each variable, individually or combined with others, can affect the results. It is of the utmost importance to evaluate each component of the process and their interactions, such as solvent, sample, power, frequency, and intensity, temperature, time, and make of the equipment.

##### Extraction solvent

Without a doubt, the choice of solvent is the primary variable in any extraction method. The extraction solvent should be chosen based mainly on the solubility and intensity of the interactions with the matrix. The characteristics of the solvent to be observed are, among others, polarity, pH, viscosity, surface tension, vapor pressure, melting point, boiling point, density, specific gravity, as well as the effect on purity, and activity of the extracted compound (Mason, and Lorimer, 2002). These factors should be thoroughly thought out, mainly because they decrease the cavitation threshold, disfavoring the removal of the compounds from the matrix (Mason, and Lorimer, 2002).

Consideration should also be given to the extraction parameters and their suitability for the solvent, the intermediate and final products to be used, and how the solvent can react with the target compounds under extraction conditions. An important factor is the biochemical and physicochemical properties of the solvents because they define the nature of the medium in addition to interacting with the treated material and extracted compounds. The possible changes that can occur in the solvents during the extraction process can have significant effects on the stability of the flavonoids and the efficiency of the treatments (Dzah et al., 2020).

A solvent with low vapor pressure, at the adjusted temperature, facilitates cavitation increasing the effects of ultrasound in the process. In contrast, viscous solutions, such as oils, increases the amplitude of the waves, hindering the propagation of ultrasound, and mechanical effects on the sample caused by the cavitation (Santos et al., 2009; Flannigan and Suslick, 2010).

In general, organic solvents (methanol, ethanol, acetonitrile, petroleum ether, acetone), water, and mixtures of these solvents are used for the removal of flavonoids from plant matrices, such as herbs, industrial residues, stems or plant seeds. Organic solvents such as ethanol, methanol, acetone, and isopropanol, mixed with varying proportions of water, have been widely used to extract flavonoids from plant sources using UAE. There are extractions with 100% of either organic solvent or water used for extraction. In some studies, extraction solvents can be acidified to preserve sensitive flavonoids from oxidative degradation (Dzah, 2014). The acids produce hydrogen ions (H+) that stabilize free radicals that may be produced during ultrasonication (Dzah et al., 2020). Several studies have shown that due to the polarity of flavonoids, organic solvents, such as methanol, are more efficient for their extraction. Non-toxic and biodegradable alternatives, such as ethanol, are being explored to some extent in extraction methods to reduce the impact of organic solvents on the environment while providing similar, or even superior performance (Fu et al., 2019). Ionic or eutectic solvents containing acids citric and lactic acid and multiphasic systems, such as cloud point extraction, are also new alternatives to toxic solvents (Vankar and Srivastava, 2010; Ekezie et al., 2017; Cunha and Fernandes, 2018; Biata et al., 2019)

The extraction performance can also be affected by the solvent pH by altering the ionic strength, which affects the solubility of the compounds and their interactions with the sample matrix. Several studies have evaluated the optimum pH to extract flavonoids from plant matrices. A recent report (Mai et al., 2020) investigated the influence of the pH of the solvent on the recovery of Euonymus alatus flavonoids and suggested that recoveries increased in acidic pH (2.5–3.5) and decreased at higher pH. Another report, which in this case evaluated polyphenols, indicated that its extraction from pomegranate peel is affected by the solvent's pH, with the best results being observed in acidic medium (Motikar et al., 2020). On pH above 7,0, lower extraction yields were recorded.

For the ultrasound-assisted extraction of bioactive compounds from the Citrus reticulata bark, slightly acidic electrolyzed water (pH 6.20) produced the best results for the extraction of total phenolic compounds. Still, higher yields of flavonoids were reported with acid electrolyzed water pH 3.24 (Soquetta et al., 2019).

Another example of the influence of the pH was reported for the ultrasound-assisted extraction of polyphenols from Satsuma mandarin leaves, where a higher yield of total flavonoids was observed at pH 2 in water. The highest amounts of total phenolic compounds and total flavonoids were achieved in acidic media (Cigeroglu et al., 2017).

The reports available in the literature suggest that higher flavonoids yields are usually produced in acidic medium. For polyphenols, this trend can be explained by the fact that an acidic pH supports the cleavage of phenolics bound to proteins and carbohydrate polymers (Ilbay et al., 2014). With a low pH value, phenols are protonated that take the hydrophobic nature to molecules that interact more strongly with the hydrophobic micellar surfactant and, therefore, readily penetrate the micelles (El-Abbassi et al., 2014). At a higher pH, phenols are deprotonated, and their ionic characteristics increase, leading to a decrease in the solubility of hydrophobic phenolic compounds in micelles due to the higher activity of protons. Thus, the amount of phenols extracted increases with the reduction of the pH (Gortzi et al., 2008; El-Abbassi et al., 2014).

##### Sample

Depending on the target compounds, the sample may be fresh or dry (plants, oleaginous, seeds, yeast, algae, among others), and the structure, moisture, plasticity, and composition of the material will entail the recovery of compounds from the sample matrix. Thus, the preparation of the sample matrix before extraction is of paramount importance, especially because some compounds are sensitive to the processes of preparation, such as drying, homogenization, and sifting. In addition to preserving the matrix compounds, the sample preparation also ensures the extraction efficiency as it can eliminate interferences, increase the concentration of the analyte in the mixture and provide the optimum particle size (Rostagno et al., 2009).

The ratios between sample quantity and solvent, as well as particle size, are also factors that should be taken into consideration to maximize extraction yield because they influence the cavitation phenomena and final concentration of the extracts (Vilkhu et al., 2011). It has been suggested that a proportion between 1:5 and 1:10 sample/solvent (solid vegetal material) for ultrasonic bath extraction is suitable for the recovery of bioactive compounds from plants (Vinatoru et al., 2017). Such high ratios may be adequate when considering the production of a concentrated extract. Still, when the objective of the extraction is sample preparation for quantitative analysis of flavonoids, a higher solvent amount (1:50, 1:100, or even higher) may be required to ensure that target compounds were removed entirely from the sample matrix. The ranges of the solid / solvent ratio are reported in the literature and it is often not indicated whether it is on a dry or wet basis. It is understood that it usually refers to fresh and not dry material. In the case of material that has been dried, it is necessary to hydrate the matrix to allow the solubilization of the compounds of interest and consequently it may be necessary to increase the ratio.

Higher efficiency can be achieved using sequential extraction processes as in each extraction, as the fresh solvent will be available and will improve solubility. Still, additional steps will be required between extractions, such as centrifugation or filtration.

Particle size is also a factor that influences the efficiency of the UAE. It should be evaluated according to the matrix, and as a function of the compounds to be extracted. In general, small particles remain on the solvent surface and are not affected by cavitation bubbles, while large particles decrease the permeability or diffusion of solvent in the sample (Khan et al., 2010). A study developed for Arecanut polyphenol extraction tested particle sizes between 841, 425, 250, and 180 μm, and the highest recoveries were obtained with a particle size of 250 μm (Chavan and Singhal, 2013). Another study using UAE for orange peel flavone extraction tested particle sizes between 0.5, 1.0, 1.5, 2.0, and 2.5 cm^2^, with the best yielding particle size being 2 cm^2^.

##### Ultrasound power, frequency, and intensity

Ultrasound power directly affects the cavitation and shear forces in the extraction medium. As ultrasound power increases, so does the cavitation and its mechanical effects as well as mass transfer of compounds from the sample matrix to the solvent. However, excessive power can negatively affect the extraction process due to the degradation of target compounds, reducing yields. To fully explore UAE as an intensification technique, it is required to adjust power considering sample moisture, the temperature of the medium, and solvent used (Wei et al., 2010).

Another critical parameter in UAE is frequency. The frequency used for the extraction of bioactive compounds from natural products usually ranges between 20 and 120 kHz. There are some reports that frequency can modulate the removal of different compounds from the sample matrix (Machado et al., 2019). In the case of phenolics from grapes lower frequency of 40 kHz provided higher yields than 120 kHz (González-Centeno et al., 2014). On another recent report, a higher yield of phenolics from pomegranate peels was also achieved with lower frequency (37 kHz). Still, higher antioxidant capacity was observed in the extracts obtained with higher frequency (80 kHz), indicating that frequency can affect the composition of the extract.

High frequencies do not allow the process of cavitation to happen fully, because they decrease the time of expansion of the bubbles, thus reducing the size and impact of these in the sample (Mason, and Lorimer, 2002). On the other hand, in low frequencies, the bubbles are in smaller quantities, but with larger diameters, which assists the physical effects generated in the sample, such as the transfer of masses between the sample and the solvent (Esclapez et al., 2011).

It is also essential to consider ultrasound intensity, which is the energy emitted per second per area of the emitting surface, being directly connected with the amplitude of the transducer and the sound wave. Thus, the higher the ultrasonic intensity, the greater the amplitude, and the better the extraction efficiency. Higher amplitudes are associated with the more significant collision between the bubbles originating from the cavitation and the sample. However, very large amplitudes can also lead to the rapid deterioration of the ultrasonic transducer, leading to liquid agitation and not to the cavitation phenomenon. Thus, attention should be paid to the amplitude, especially considering the solvent, where high amplitudes are suitable in more viscous solvents such as the oils (Tiwari, 2015; Machado et al., 2019).

##### Temperature and extraction time

The temperature of the medium should be closely related to the properties of the solvent since the temperature increase causes a decrease of the viscosity and surface tension of the solvent but increases the vapor pressure. Increased vapor pressure decreases the effectiveness of the cavitation process and leads to lower extraction efficiency.

The vapor pressure of the liquid influences the cavitation process, and lower vapor pressure solvents are more advisable in UAE extractions because they induce a more significant collapse between cavitation bubbles. Thus, solvents with high vapor pressure and, consequently, high boiling temperatures, do not fully explore the potential of UAE due to reduced cavitation (Flannigan and Suslick, 2010).

High temperature, however, also facilitates the increase of cavitation bubbles, increasing the contact area and diffusion between solid and solvent. Thus, for better effects of ultrasound associated with cavitation, medium to low temperatures are indicated (between 20 and 70°C), depending on the sample, and especially for thermosensitive ones (Palma et al., 2013; Pasrija and Anand haramakrishnan, 2015).

Maran et al. (2017) studied the effect of extraction temperature, among other parameters, on the recovery of anthocyanin, flavonoids, and total phenolics of Nephelium lappaceum bark extracts obtained through the UAE. It was observed that the yield was increased due to the increase in porosity of the material, more significant solvation, and mass transfer when the extraction temperature increased.

On the other hand, the required extraction time of the process will depend on several factors. The overall extraction kinetic curve can be divided into three stages: constant extraction rate (CER), where compound are more easily extracted from the sample matrix; falling extraction rate (FER), where compounds being extracted present some interactions with the matrix, hindering their removal; and diffusional controlled (DC), where compounds depend on diffusion to be removed (Palma et al., 2013).

When considering the production of extracts, the extraction time is usually determined by the conditions that are most favorable to the mass transfer from the matrix to the medium, generally between CER and FER part of the extraction curve. However, the exact extraction time will depend on several economic factors and manufacturing costs, but especially the raw material cost (Palma et al., 2013). In contrast, for analytical purposes, it is necessary to ensure that target compounds where completely removed from the sample, which is usually done by overextending the extraction time.

Applications for obtaining concentrated extracts of flavonoids, which usually exploit the first stages of the extraction process (CER and FER), can also explore the benefits of the use of ultrasound. There is a more significant potential of this technique in the preparation of samples for quantitative analysis since ultrasound has a substantial effect on diffusion and can accelerate the final phase of the diffusion-controlled extraction process (CD).

There are other important aspects associated with extraction time, including the amount and type of solvent used, the amount and characteristics of the sample (protein content for example), temperature, flow rate (in dynamic extractions), ultrasound intensity and frequency, potential degradation of target compounds, among others, making this one of the most challenging techniques to be optimized. Usually, extraction time is determined experimentally on a case by case basis.

#### Applications of UAE to Flavonoids From Natural Products

During the last decade, several UAE methods have been developed, and a significant number of applications for the recovery of flavonoids can be found in the literature. Some of these applications are shown in Table 2. The use of ultrasound has grown in recent years. In most cases, the produced results indicate that it accelerates the extraction process and decreases the amount of solvents used (Zhang L. et al., 2009; Oniszczuk and Podgórski, 2015). The most used solvents for the extraction of flavonoids are ethanol, mixtures with water at different proportions, and natural deep eutectic solvents (NADES), which are based on their ability to solubilize moderately polar flavonoids with a relatively low cost and environmental impact. Regarding ultrasound, the most used type of equipment is the ultrasonic bath operating at 40 kHz with fixed power. Specific conditions, such as temperature and extraction time, vary significantly due to the particularity of the composition of each raw material.

**Table 2:** Experimental conditions used for extraction ultrasound flavonoids from natural products.

**Sample**	**Solvent (%)**	**Power (W)**	**Frequency (kHz)**	**Temperature (°C)**	**Time (min)**	**Sample/volume (g:mL)**	**Equipment**	**Target compounds**	**Yield or recovery**	**Reference**
Cocoa Shells (*Theobroma cacao*)	70–90 ethanol	296	40	45–65	30–60	1:50	Bath	Total flavonoids	7.47 mg/g	Md Yusof et al., 2019
Basil leaves (*Ocimum tenuiflor*)	30–70 Ethanol	50	30	40	5–15	1:40	Ultrasonic processor	Total flavonoids	6.69 QE mg/g	Upadhyay et al., 2015
Eucommia leaves (*Folium eucommie*)	30–50 ethanol	850	59	5	5–75	1:50	Bath	Total flavonoids	41%	Huang et al., 2009
Guava leaves (*Psidium guajava*)	Water	250–450	–	0–80	5–45	5:200	Bath	Total flavonoids	Not mentioned	Li et al., 2019
Sophora leaves (*Sophora flavescen*)	40–80 methanol	120	40	20–80	20–80	1:26	Bath	Trifolirhizin Formononetin Isoxanthohumol Maackiain Kurarinone	2.570 mg/g 0.213 mg/g 0.534 mg/g 0.797 mg/g 3.091 mg/g	Zhou et al., 2019
Rugosa rose fruit (*Rosa rugosa* Thunb.)	50–95% ethanol	200	40	30–50	30–50	1:20	Bath	Individual flavonoids	54.32–75.23 mg/g	Um et al., 2018
Hawthorn seed	55–85% ethanol	40	40	55–75	30–50	1:22	Bath	Total flavonoids	16.45 mg/g	Pan et al., 2012
Flower (*Citrus aurantium* L. var. amara Engl)	40–80% ethanol	200	–	30–80	10–80	1:20	Bath	Total flavonoids	1.87%	Yang et al., 2010
Grapefruit (*Citrus paradisi* L.)	20–100% ethanol	100	40	4–70	5–48	1:8	Bath	Total flavonoids	50%	Garcia-Castello et al., 2015
Curry leaf (*Murraya koenigii* L.)	40–80% methanol	880–150	–	40–80	20	1:20	Bath	Naringin Epicatequin Catequin Quercetin Myricetin	0.203 mg/g 0.678 mg/g 0.325 mg/g 0.350 mg/g 0.703 mg/g	Ghasemzadeh et al., 2014
*Canna indica*	Methanol. ethanol. ethyl-acetate. acetone and water	100–500	20	25–30	5–20	20:50	Bath	Total flavonoids	0.3409 g/g	Srivastava and Vankar, 2010
Wine less	NADES	380	37	35	5–45	0.1:1	Bath	Anthocyanins	Not mentioned	Bosiljkov et al., 2017
Dittany (*Origanum dictamnus*), fennel (*Foeniculum vulgare*), manjoram (*Origanum majorana*), sage (*Salvia officinalis*) and mint (*Mentha spicata*)	NADES	140	37	80	90	0.1:15	Bath	Total flavonoids	109.67 mg GAE g−1 dw	Bakirtzi et al., 2016

### Microwave-Assisted Extraction (MAE)

#### Fundamentals

Currently, novel green extraction techniques with efficient and rapid process have appeared to correct the limitations of conventional extraction methods, such as the vast quantities of solvents used, the high temperatures applied, or the long extraction times needed (Dahmoune et al., 2014).

**Figure 3:**
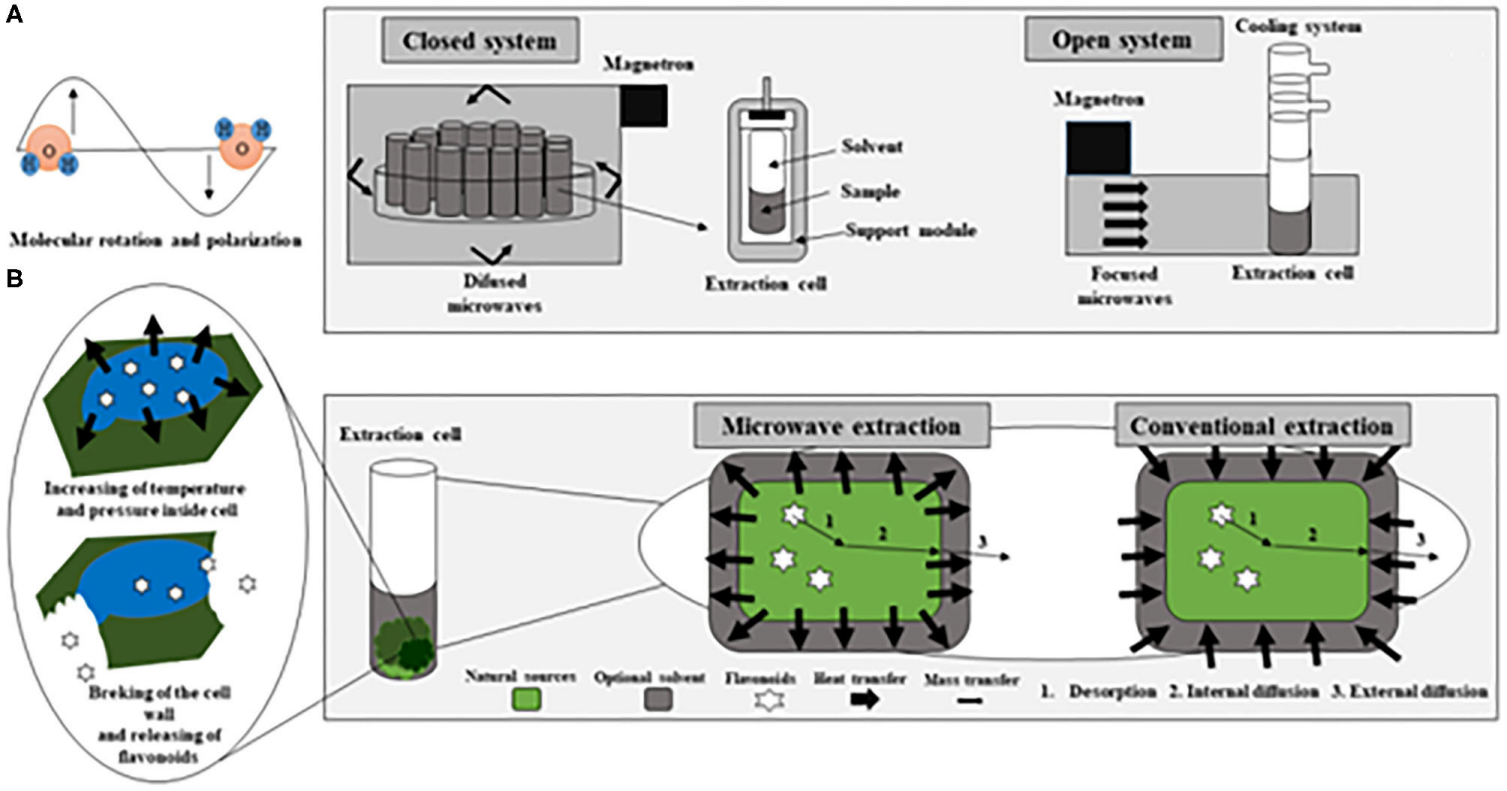
Representative diagram of a microwave extractor in both closed and open systems **(A)** adapted of Camel (2001); Representative diagram of the conventional heating and microwave heating **(B)** adapted of Rosa et al. (2018) and Destandau et al. (2013).

Microwaves are non-ionizing electromagnetic (EM) waves located between the radio-frequency range at the lower frequency and infrared at the higher frequency in the electromagnetic spectrum within the frequency band of 300 MHz to 300 GHz. In this extraction technique, the microwave energy is delivered through polar components interactions to generate heat by conversions of electromagnetic into thermal energies (Pimentel-Moral et al., 2018b). This conversion occurs via two mechanisms: by dipole rotation, i.e., through reversals of dipoles and by ionic conduction, i.e., by displacement of charged ions present in the solute as well as the solvent (Dean et al., 1995). When microwave energy absorption occurs, the conversion of electromagnetic energy into heat depends on the relation between the dielectric loss factor (ε″) and the dielectric constant (ε′) for a given material (Mello et al., 2014). This relation is known as the dissipation factor (or loss tangent, tan δ). MAE heats all the sample fluid, allowing the extraction solution (solvent and sample) to reach the desired temperature more rapidly. It avoids the thermal gradient caused by conventional heating (Biesaga, 2011), which increases the risk of degradation of thermolabile bioactive compounds (Wu et al., 2001). Therefore, the improvement in extraction yields is not only produced by the increase in temperature within the extraction medium, but also by the effect of microwave electromagnetic radiation on vibrations of both types of bonds (extraction solvents and the analytes to extract) (Ameer et al., 2017). Concerning the release of the compounds from the matrix, during microwave heating, a considerable amount of pressure builds up inside the biomaterial, improving the porosity of the matrix, which allows better penetration of extracting solvent through it (Kratchanova et al., 2004). Figure 3 represents a microwave extraction system, both in a closed system and in an open system, in addition to diagramming the difference between conventional heating and microwave heating (Camel, 2001; Destandau et al., 2013; Rosa et al., 2018).

#### Influential Parameters in the Extraction Process

The degradation of flavonoids can be caused by several factors such as light, air, time, and temperature. Furthermore, the efficiency of MAE depends to a great extent on the selection of the operating conditions and the parameters that affect the extraction mechanisms and performance. The choice of an extraction method is based on the highest recovery of the targeted compound, retention of the required properties of the compound ease of application of the extraction method with available resources, and the properties of the targeted flavonoid compound. Various factors influence the performance of the MAE, such as the nature of the solvent, the ratio sample amount: solvent volume, the extraction time, the microwave power, the temperature, among others (Chan et al., 2011).

##### Extraction solvent

As mentioned above, the dielectric constant and the dissipation factor are two critical parameters involved in MAE. Therefore, it is essential to choose solvent mixtures to modify the dielectric constant until obtained suitable characteristics for the extracted sample. On the one hand, solvents like ethanol, methanol, or water can absorb microwave energy due to their high dielectric constant and dielectric loss, which can lead to a faster rate of heating of the solvent concerning the plant material (Zhang F. et al., 2009). On the other hand, for the extraction of thermolabile compounds, a solvent combination with relatively lower dielectric properties can be used to ensure that the solvent temperature will remain lower to cool-off the solutes once they are liberated into the solvent (Routray and Orsat, 2012).

The polarity of the solvent and the solubility of the targeted compound in the solvent must also be considered. There is no standardized solvent composition for all since it is difficult to establish general rules, so the best solvent varies with each targeted compound (Xiang and Wu, 2017). In general, the solvent most used for polar flavonoids extraction is a mixture of water and organic solvents. Within these, methanol is highly toxic and is not practical for use in the processing of food and pharmaceutical products. It is generally used in analytical applications in different proportions depending on the compounds to be extracted (Chen et al., 2008). The most commonly used is ethanol, as it is a green solvent with low toxicity. As mentioned above, adding a certain amount of water to the ethanol solvent has been shown to improve extraction efficiency. However, as can be seen in Table 3, the best extraction yields are obtained with different concentrations of ethanol (35–90%) in aqueous solution, according to the literature consulted. The presence of water would improve the mass transfer between the solid and the liquid by increasing the permeability of the matrix of the plant, thus improving heating efficiency (Zhang et al., 2007; Zhong et al., 2016). However, the optimal percentage of water-ethanol will depend on the characteristics of the matrix, the extraction conditions (power, temperature, time, among others), and the compounds to be extracted. For less polar flavonoids, such as aglycones of isoflavones, flavanones, methylated flavones, and flavonols, solvents used also include chloroform, acetone, dichloromethane, diethyl ether, hexane or ethyl acetate (Grigonis et al., 2005). Some specific examples for diverse matrices using the mentioned above solvent are shown in Table 3.

**Table 3:** Some representative applications involving the use of MAE for the recovery of flavonoids from natural sources.

**Sample**	**Solvent (%)**	**Power (W)**	**Extraction volume (mL)**	**Temperature (°C)**	**Time (min)**	**Sample/volumen (g:mL)**	**Target compounds**	**Quantification method**	**Yield or recovery**	**Reference**
Grapes skin (Tintilla de Rota)	40:60% methanol:water	500	25	100	5	2:25 g DW:mL solvent	Individual Anthocyanins	HPLC-DAD, using malvidin-3-glucoside as standard	1.86 mg standard/g DW	Liazid et al., 2011
*Morus alba* L. leaves	60:40% etanol:water	560	250	100	5	1:15 g FW:mL solvent	Total flavonoids	NaNO_2_- Al(NO_3_)_3_-NaOH colorimetric assay, using rutin as standard	2.5%	Li et al., 2009
*Platycladus orientalis* L. leaves	80:20% methanol:water	80	5	–	5	1:5 g DW:mL solvent	Total flavonoids	Dynamic MAE coupled with on-line derivatization and UV–vis detection, using quercitrin as standard	98.5% (w/w)	Chen L. et al., 2007
*Gordonia axillaris*	36.89:63.11% ethanol:water	400	15	40	71.04	1:29.56 g DW:mL solvent	Total flavonoids	Method described by Lamaison and Carnet (1990) with some modifications, using as standard solution	3.11 mg standard/g DW	Li et al., 2017
Tomato (Round tomato)	100% water	200	20	60	20	45:1,000 g DW:mL solvent	Individual flavonoids	HPLC, using external standards for each flavonoid	6.78–11.7 mg standard/g DW	Pinela et al., 2016
*Morus nigra* L.	70:30% ethanol:water	500	125	35	10	1:50 g DW:mL solvent	Total flavonoids	Aluminum chloride method, using quercetin as standard	2.35–2.83 mg standard/g DW	Koyu et al., 2018
*Radix Astragal* roots	90:10% ethanol:water	1,000	50	110	25	1:25 g DW:mL solvent	Individual flavonoids	HPLC-UV, using external standards for each flavonoid	1.190 mg standard/g DW	Xiao et al., 2008
Apple (*Malus domestica*) roots	60:40% ethanol:water	1,500	20	100	20	0.1:20 g DW:mL solvent	Individual flavonoids	HPLC, using external standards for each flavonoid	17.1 mg standard/g DW	Moreira et al., 2017
Black rice (*Oryza sativa* cv. Poireton) husk	67.34:32.66% ethanol:water	640	–	49.5	0.5	1:40.79 g DW:mL solvent	Total flavonoids	Aluminum chloride method, using catechin as standard	0.0304 mg standard/g DW	Jha et al., 2017
Green tea (*Camellia sinensis* L.)	100% water	100	200	50	15	1:20 g DW:mL solvent	Total and individual catechin	Folin-Ciocalteu (Total) and various spectroscopic analysis along with their respective catechins (individual)	8.80%	Kalai and Ignasimuthu, 2018

The pH of the solvent can also significantly affect the efficiency of the extraction steps. The effect of pH on the flavonoids extraction is closely related to their structure. Given the broad class of existing flavonoids, authors have reported different optimal pHs for its extraction. Some authors (Xin et al., 2008; Xiangnan et al., 2014; Bouras et al., 2015) have shown that alkaline extraction was more efficient in recovering flavonoids than acidic solvent extraction (pH 12 > pH 7 > pH 2). Specifically, Bouras et al. (2015), founded that flavonoid compounds [naringenin, (+)-catechin, (–)-epicatechin, (–)-epigallocatechin] increased ~1.25 times with the presence of 0.01 M sodium hydroxide compared to pure water, when extracting flavonoids from *Quercus* bark using MAE. This behavior is linked to the fact that basic pHs remove tannins, phenolic acids, and other polyphenols linked by ester bonds, reduces bark extract viscosity, and increases the reactivity of the extract, increases the accessibility of solid residue by removing the lignin physical barrier, and damages the cell structure. Other authors showed better extractions with acidic solvents. It happens when anthocyanins are studied, a class of flavonoid which have a stable conformation, the cation flavilium, at pH from 1.0 to 3.0. In cases where the use of acid is necessary, low concentrated acids and mainly hydrochloric acid can be used until a pH between 1 and 4.5 is achieved (Yang and Zhai, 2010; Teng et al., 2013).

In addition to common solvents, ionic liquids (ILs) have been reported as eco-friendly solvents for the MAE of several flavonoids. Room-temperature ILs, resulting from the combination of organic cations and various anions that may be liquids at room temperature, are salts with melting points of below ca. 100°C. The main ILS used in the literature are: 1-butyl-3-methylimidazolium chloride [[bmim]Cl], 1-butyl-3-methylimidazolium bromide [[bmim]Br], and 1-butyl-3-methylimidazolium tetrafluoroborate [[bmim]BF_4_] between others. For the synthesis of [bmim]Cl and [bmim]Br, a mixture between 1-methylimidazole and the corresponding halogenoalkane in a 1:1.2 molar ratio is employed. [bmim][BF_4_] is prepared by the reaction of [bmim]Cl and NaBF_4_ at the same molar ratio. All the solutions are prepared at different concentrations with deionized water (Liu et al., 2005). Specifically, ILs have extended into areas of analytical chemistry and liquid-liquid extraction, and show excellent solvent properties: negligible vapor pressure, a wide liquid range, good thermal stability, tunable viscosity, miscibility with water and organic solvent, and good solubility and capacity of extraction for several organic compounds (Deng et al., 2006). The ionic liquids employed and their concentrations (usually between 1.5 and 3 mol L^−1^) have great influence in the MAE extractions of the flavonoids. Concerning the type of IL employed, the extraction yields of flavonoids are largely dependent on the anions for the same class of ILs, highlighting Br^−^, Cl^−^, ${\text{BF}}_{4}^{-}$, ${\text{SO}}_{4}^{2-}$, $\text{N}{\left(\text{CN}\right)}_{2}^{-}$, H_2_${\text{PO}}_{4}^{-}$, ${\text{PF}}_{6}^{-}$, CF_3_${\text{SO}}_{3}^{-}$, and CF_3_${\text{CO}}_{2}^{-}$, due to the anion-dependency of the solubilities of analytes in ILs (Du et al., 2009). Furthermore, ILs which have cationic moieties with an electron-rich aromatic π-system produced stronger interactions with solute molecules capable of undergoing polarity, π-π and n–π interactions (Du et al., 2007). Concerning the ILs concentration, Du showed that the yields of the extraction of polyphenolic compounds from *P. guajava* leaves and S. china tubers increased with the increase in the concentration of [bmim]Br. This behavior is due to the solvation power and multiple interactions of [bmim]Br and its capacity to change the dissipation factor of the solution and improved the transfer efficiency of microwave energy.

##### Sample: volume ratio

Another factor that affects MAE is the relationship between the amount of sample and the solvent volume. The objective should be to minimize the use of solvent and maximize the yields. On the one hand, high volumes of solvent require more microwave energy due to microwave radiation would be absorbed by the solvent. This additional power could cause the solvent heating rate to increase drastically, which results in the thermal degradation of the bioactive compounds (Spigno and De Faveri, 2009; Alara et al., 2018a). On the other hand, low volumes of solvent promote the mass transfer barrier, since the distribution of the active compounds is concentrated in certain regions, which limits the movement of the compounds outside the matrix of the cell (Hao et al., 2002). An optimum ratio sample amount: solvent volume assurance uniform and effective heating (Zhou and Liu, 2006). Some authors indicate that the ratio most used for the extraction of this type of compound is around 1:30 g/mL (Zhao et al., 2018). For much of the matrices shown in Table 3, ratios around this value were used.

##### Microwave power

The plant cell walls tend to absorb microwave energy and cause an increase in internal superheating resulting in cell disruption that facilitates leaching out of flavonoids from the samples and so that the analytes can diffuse and dissolve in the solvent (Bouras et al., 2015). In general, the extraction performance increases with higher microwave power, up to 500 W (1 g of leaf powder) (Mandal and Mandal, 2010). For many of the matrices shown in Table 3, microwave powers up to this value were used. In any case, it is necessary to take into account not only the applied power but also its relation to the sample mass (power density). However, very high power can lead to lower yields, which can be attributed to the heat generated by the microwave energy causing the disintegration and thermal degradation of the total flavonoid content in the sample (Dahmoune et al., 2014; Alara et al., 2018b). Alara et al. (2018a) obtained an improvement in the yields of the total flavonoids as the level of microwave power increased from 400 to 500 W (10 g of leaf powder). However, the yields declined with power above 500 W, with the lowest values observed at 600 W.

##### Extraction temperature

Concerning the extraction temperature, it is an essential factor to be considered both in open system extraction and in closed system extraction. High temperatures cause a decrease in viscosity and surface tension, which allows better penetration of the solvent into the sample matrix. Also, it increases the molecular movement by accelerating the mass transfer of intracellular bioactive compounds from the plant matrix (Zhao et al., 2017). However, above 100°C, the extraction yield decreases by breaking down the molecular structure of the bioactive compounds (Pimentel-Moral et al., 2018b). However, this temperature limit varies with the type of compounds to be extracted, since the degradation temperature of each compound is different. The number and type of substituents present in the aromatic ring, as well as the position, influence stability. A smaller number of substituents increase flavonoid stability.

Additionally, when the compounds have several substituents on the ring, the hydroxylates are more readily degradable than the methoxylated ones (Routray and Orsat, 2012). Also, the sugar portion stabilized the flavonoids during the extraction process. Several researchers use temperatures around 60°C since it is sufficient to extract the compounds of interest without causing their degradation (Jin et al., 2017; Xiang and Wu, 2017). However, there are other authors that for certain compounds use higher temperatures, even higher than 100°C (Pinela et al., 2016).

Finally, it is also essential to have in mind the relationship between temperature and extraction time. With increasing extraction time, the extraction yields will firstly increase and then will decrease, probably because over-exposure to microwave temperature may lead to thermal degradation of bioactive compounds. So, it is necessary to reach equilibrium between the applied extraction time and temperature.

##### Extraction time

The yields of extracts tend to increase when the extraction time increases. However, at prolonged time of exposure to microwave radiation, even at low temperatures or low operating power, the risk of thermal degradation of polyphenols chemical structures is inevitable (Wang et al., 2010). Therefore, there will be an irradiation time limit for each of the bioactive compounds present in the matrix in which quality yields can be obtained (Yedhu and Rajan, 2016). For this reason, the extraction time of the MAE can vary from a few minutes (Carniel et al., 2017; Liang et al., 2017) to more than half an hour in some cases (Bouras et al., 2015; Zhao et al., 2018). If a longer extraction time is required, extraction cycles can be used, which consists of the extraction of the sample in several successive stages in order not to use severe conditions (Zhao et al., 2018).

##### Plant matrix characteristic

Besides the operating conditions discussed in the previous sections, the characteristics of the sample also affect the performance of MAE. A critical factor in the recovery of flavonoids is the particle size of the sample. Fine powders can improve the extraction process by providing a large surface contact area between the solvent and sample. Sample diameter reduction also ruptures cell walls, which increase the penetration of the microwave radiation. Thus, they are directly exposed to extraction solvent as well as a shorter distance for the transfer of target compounds through cell walls to solvent, thus increasing the extraction yields (Kong et al., 2010). For the production of fine powders, the sample preparation steps are essential and include drying, milling, grinding, and homogenization of the sample before the extraction for optimum extraction yield. The only problem associated with the use of fine particles is that it would cause difficulty in separating the extract from the residue, and probably, additional clean up steps may have to be employed (Chan et al., 2011).

Furthermore, to this essential step, other pretreatments can be applied to the sampled. For example, samples treated by solvent for 90 min before extraction can enhance the heating efficiency of MAE, promote the diffusion and improve the mass transfer of active compounds to the solution (Pan et al., 2003). Also, the dried sample matrix pretreated with water helps localized heating of microwave. In this sense, apart from the particle size, the solvent pretreatment has considerable effects on the sample matrix for efficient extraction.

#### Applications of MAE to Flavonoids From Natural Products

MAE has been applied successfully in the development of methods for the extraction of bioactive compounds of interest, both polar and non-polar, in multiple matrices. Specifically, MAE has been increasingly recommended in the last few years as an excellent method for the lab-scale, precision, and quantification studies of flavonoid samples. However, scale-up based on microwave extraction for the large-scale production of flavonoids is still under research and development (Orsat and Routray, 2017). Furthermore, in comparison with UAE, the extraction technique aforementioned, it has been observed that microwave extraction obtains similar or higher yields in the case of flavonoids (Gao and Liu, 2005; Chen X. J. et al., 2007). For example, Casazza et al., 2010, achieved higher amounts (46.7 mg GAE/g DW) of total flavonoids employing MAE than employing UAE (39.5 mg GAE/g DW) in *Vitis vinifera* wastes. Similar behavior was obtained by Gao and Liu (2005) (yield of flavonoids of 4.1% using MAE and 3.5% using UAE) from cultured cells of *Saussurea medusa* Maxim.

MAE in a closed system is quite similar to the PLE technology, as the solvent is heated and pressurized in both systems (Camel, 2001). According to Søltoft et al. (2009), the efficiencies of PLE was comparable and at the same level as MAE for the extraction of flavonoids in onions. Specifically, about 500 μg quercetin g^−1^ dry weight was achieved, showing a significantly better PLE extraction in the case of the quercetin-4-glucoside compound. For example, extraction yield and total flavonoid content under optimal PLE conditions were higher than MAE (56 ± 2%, 6.5 ± 0.2 mg Eq quercetin/g and 26 ± 2%, 2 ± 0.5 mg Eq quercetin/g dry leaf, respectively) for the extraction of bioactive compounds from *M. oleifera* leaves. However, the antioxidant activity was higher in MAE extracts than PLE (16 ± 1 Eq Trolox/100 g dry leaf and 12 ± 2 mmol Eq Trolox/100 g dry leaf) (Rodríguez-Pérez et al., 2016). Specifically, these authors highlighted that the extraction method should be selected depending on the target compounds to be isolated. According to their results, PLE was more suitable for extracting phenolic compounds having a higher number of hydroxyl-type substituents (kaempferol diglycoside and its acetyl derivatives or malonyl, hydroxyl, or acetyl glycosylated of quercetin) and those that are sensitive to high temperatures (glucosinolates or amino acids). Therefore, according to these authors, MAE, and PLE seem to be good options to extract bioactive compounds such as flavonoids, with little difference in their yields and due, in many cases, to the target extraction compounds rather than the technique itself. It should be noted that powers used in microwave pretreatment are lower than in PLE because high microwave power values result in lower total dry extract yields due to degradation of the target compounds (Kovačević et al., 2018). However, due to the few articles found in the bibliography where both techniques have been applied under the same matrices and conditions, no general conclusion can be reached.

According to the comparison with the other extraction techniques previously described, although SFE is a promising method in terms of extraction performance, some of its operational attributes are less versatile than in the case of other techniques, such as MAE. For example, Mustapa et al. (2015) obtained a low yield for the extraction of compounds from the medicinal plant Clinacanthus nutans Lindau using SFE, with a value of only 3.19% g/g DM; compared to MAE that obtained an amount of 17.39% g/g DM. The lower SFE yield is probably due to the non-polar nature of the carbon dioxide solvent employed in SFE, which is unfavorable for extracting the abundant polar compounds present in this sample, such as various flavonoids. In supercritical extraction, CO_2_, the primary solvent used, has low polarity. For these reasons, for the removal of polar compounds, polar solvents must be added, which decreases the extraction selectivity. Furthermore, a high concentration of a polarity modifier can lead to a temperature of the mixture lower than the critical temperature, which cancels the benefit of using supercritical extraction (Camel, 2001).

Bioactive flavonoids are mainly present in edible parts but can also be present in other non-edible parts of the plants, including leaves, stem, and root. Where in some instances, the amount of these compounds is comparable or higher than the amount reported in edible parts of the same plant. It has been widely employed in the extraction of a different kind of matrix, i.e., fruits like grapes and apples, vegetables such as onions and tomatoes, different types of leaves like olive leaves and green tea leaves, or even in blackcurrant marc, among others. It can also be used directly on the fresh sample or after a drying and lyophilization process. Extraction of flavonoids with optimized MAE technique, could not only contribute to the nutraceutical industry (health-promoting foods) but could also aid in decreasing by-product pollution, in the reduction of energy consumption and the creation of new sources of income (Sillero et al., 2018). Table 3 lists examples of successful MAE applications for the extraction of flavonoids in different matrices in recent years.

### Pressurized Liquid Extraction

#### Fundamentals

Pressurized liquid extraction (PLE) has also been referred to in the literature as pressurized fluid extraction (PFE), accelerated solvent extraction (ASE), and pressurized solvent extraction (PSE). It is an automated technique for extracting solid samples with liquid solvents. When water is used as an extraction solvent, different terms can be used to define the method, including hot water extraction (HWE), subcritical water extraction (SWE), high-temperature water extraction (HTWE), hot water extraction pressurized (PHWE), liquid water extraction or superheated water extraction.

PLE was introduced in 1995 by Dionex Corporation as an alternative to maceration, percolation, sonication, ultrasound, microwave-assisted, soxhlet extraction, and other extraction techniques such as supercritical fluid extraction (SFE) and microwave-assisted extraction. It was initially termed Accelerated Solvent Extraction technology (ASE).

In PLE, environmentally friendly liquid solvents are used at moderated-elevated temperatures and pressures to increase the efficiency of the extraction process. Under PLE conditions, the solvent is kept in a liquid state, providing a better mass transfer of the essential compounds present in the plant matrix to the solvent, as well as the stability of the process (Evstafev and Chechikova, 2016). The increase of the temperature causes dramatic changes in the physical-chemical properties of water, enhances the solubility of the analytes, breaking matrix-analyte interactions achieving a higher diffusion rate, and especially in its dielectric constant (ε). In contrast, the increase of the pressure allows keeps the solvent below its boiling point. Dielectric constant (polarity of the solvent) is a critical parameter in affecting solute-solvent interactions, and in the case of water, increasing the temperature under moderate pressure can mainly decrease this constant. At standard conditions of pressure and temperature, water is a polar solvent with a high dielectric constant (ε = 78). Still, at 300°C and 23 MPa, the ε decreases to 21, which is similar to the value for ethanol (ε = 24 at 25°C) or acetone (ε = 20.7 at 25°C). This means that at moderated-elevated temperatures and moderate pressures, the polarity of water can be reduced considerably and can act as if ethanol or acetone were being employed (Plaza and Turner, 2015; Lachos-Perez et al., 2017; Tena, 2018).

The main advantages of PLE are the following: faster extractions (around 15–50 min), low amount solvent (15–40 mL), no extract filtration required. The main drawbacks are the high cost of the equipment and the need for a thorough optimization of variables to avoid a matrix-dependent efficiency (Wijngaard et al., 2012). PLE, either an aqueous or an organic solvent can be carried out in the static, dynamic mode, or a combination of both. In the static mode, the sample, and solvent kept for a specified time at constant temperature and pressure, whereas in the dynamic mode, the solvent flows through the sample continuously. A more detailed description of the main principles of PLE and the influence in the extraction process of different parameters that affect performance are described in the next section.

#### Operating Parameters for Pressurized Liquid Extraction

Several factors that influence the pressurized liquid extraction process, such as sample size, solvent, pressure, temperature, pH, flow rate, and extraction time, must be considered. The parameters with the most significant effect in the PLE process are the type of solvent and the temperature (Wijngaard et al., 2012).

##### Sample size

The size of the particle is a crucial factor. The reduction of the particle size facilitates contact with the solvent and the extraction of the solute from inside the matrix due to the smaller diffusion path (Mustafa and Turner, 2011). Consequently, the extraction process would result in shorter processing time due to the exhaustion of the solute within each part of the matrix.

##### Solvents

The selection of the solvent in the PLE for a solid-liquid system is significant since the desired compounds must have a high solubility in the solvent. The characteristics of the solute and solvent must be identical (Azmir et al., 2013), polar solute in a polar solvent, as well as the non-polar solute, solubilized in a non-polar solvent (Machado et al., 2015). The chemical properties of the solvent must guarantee a higher solubility of the solute, as well as its selectivity. However, achieving selectivity is a great challenge, and the scientific community has focused on research to optimize the experimental conditions to obtain a specific compound, as shown in Table 4.

**Table 4:** Survey on the applications of PLE to obtain flavonoids from natural sources using environmentally-friendly organic solvents.

**Sample**	**Solvent (%)**	**PLE conditions (mode, reactor volume, extraction time, solvent flow rate)**	**Temperature (°C)**	**Pressure (bar)**	**Target compounds**	**Yield or recovery**	**Reference**
Goldenberry	Ethanol (70%)	Dynamic, 7.5 mL, 10–60 min, 1–3 mL/min	N/I	100–200	Quercetin Rutin_hydrate Mangiferin	0.57 mg/L 1.20 mg/L 3.57 mg/L	Corazza et al., 2018
Grape marc	Ethanol (50–100%) Water-ethanol acidified	N/I, 50 mL, 220 min, 5 g/min	40–100	100	Anthocyanins	10.21 mg malvidin-3-O-glucoside/g dr	Pereira et al., 2019
Citrus by-products	Ethanol (50–99.5%)	Dynamic, 10 mL, 5–40 min, 2.37 g/min	45–65	100	Hesperidin	19.3 mg/g dry peel	Barrales et al., 2018
Passion fruit rinds	Ethanol (70–100%)	Dynamic, 10 mL, 60 min, 2.7–3 mL/min	30–60	100	Isoorientin Vicenin Orientin Isovitexin Vitexin	118 μg/g dried rind 85 μg/g dried rind 25 μg/g dried rind 34 μg/g dried rind 17 μg/g dried rind	Viganó et al., 2016
Waxy barley	Ethanol (5–20%)	Static, 100 mL, 15–55 min, 2–6 mL/min	135–175	150	Catechin, Naringinin, Morin, Rutin, and Quercetin	N/I	Benito-Román et al., 2015
*Dracocephalun kotschy*i	Methanol	Static and dynamic, 100 mL, N/I, 0.4–0.8	50–80	20–40	Quercetin Luteolin	6.13 mg/g 13.25 μg/g	Kamali et al., 2016
*Lippia citriodora* leaves	Ethanol (15–85%)	Static, 33 mL, 5–20 min, N/I	40–180	110	Luteolin-7-diglucuronide, apigenin-7-diglucuronide, chrysoeriol-7-diglucuronide, acacetin-7-diglucuronide, methyl quercetin, dimethyl kaempferol, and dimethyl quercetin	N/I	Leyva-Jiménez et al., 2018
*Moringa oleifera* leaves	Ethanol (55–100%)	Static, N/I, 60–210 min, N/I	110–140	N/I	Quercetin and kaempferol	N/I	Wang et al., 2017

The use of solvents in this context is based on the concept of green chemistry, that during the development of the extraction process is necessary minimum quantities of solvent (Ibañez et al., 2012), as well as its easy recovery and reused that unlike conventional techniques becomes less sustainable (Mustafa and Turner, 2011; Wijngaard et al., 2012). The type of solvent to be used within the must present characteristics that are less toxic to reduce the environmental and economic impact, favoring a smooth recovery (Azmir et al., 2013). Besides, the few volumes of solvents used in PLE due is a consequence of the combination exerted by high temperature and pressure, reducing the time of the process (Ibañez et al., 2012).

The most used solvents are water and ethanol for PLE, of the most popular methods of extraction according to the solvent used, there is the denomination of extraction with subcritical water, where the solvent is 100% water (Wijngaard et al., 2012). However, in the subcritical state, the application for the extraction of thermally unstable compounds, such as anthocyanins, may be limited. Whose mixtures have also proven to be more 20 effective than traditional extraction solvents, as was already evidenced in some investigations (Machado et al., 2015; Pereira et al., 2019), demonstrating that the mixture of solvents with a moderate polarity has a positive influence on the recovery of phenolic compounds (Xynos et al., 2014).

##### Pressure

The high pressure applied in the PLE is to maintain the solvent in the liquid state, being able to vary the temperature in ranges above the boiling point. The effect of the high pressure of the system gives rise to a phenomenon called penetration, which directs the solvent into the pores of the solid matrix by extracting the analytes (Wijngaard et al., 2012). The effect of high pressure is an essential factor in the changes in the physical-chemical properties of the solvent, such as density, diffusivity, viscosity, and dielectric constant. That is a particular part it improves the penetration phenomenon of the liquid phase in the solid matrix (Mustafa and Turner, 2011). In literature, it is possible to find several studies that report that pressures above of 100 bar increase the PLE yield, from a different matrix, for instance, goldenberry, grape marc, citrus by-products, passion fruit rinds, Lippia citriodora leaves (Viganó et al., 2016; Barrales et al., 2018; Corazza et al., 2018; Leyva-Jiménez et al., 2018; Pereira et al., 2019). However, in the case of flavonoid glycosides and aglycones, such as quercetin, luteolin, apigenin, narirutin, naringin, etc., it is possible to obtain high extraction yields at pressures low as 30 bar (Kamali et al., 2016; Cvetanović et al., 2018; Munir et al., 2018).

##### Temperature

The temperature is a critical factor within the PLE, and the thermal effect is of significant influence on the stability of the compounds within the plant matrix. The bioactive compounds can be selectively removed by modifying the temperature; however, the degradation of the thermosensitive compounds can take place at higher temperatures, and consequently promote the formation of undesirable compounds (Wijngaard et al., 2012; Evstafev and Chechikova, 2016; Lachos-Perez et al., 2020). The effect of the temperature generates a high solubility of the analytes extracted in the solvent because the increase in temperature reduces the viscosity resulting in an increase in the extraction rates of the analyte by diffusion to the solvent (Mustafa and Turner, 2011; Evstafev and Chechikova, 2016). However, the high extraction rates are due to a rupture of solute ligations in the matrix due to temperature (Machado et al., 2015).

Among the different studies carried out in extraction, the temperature ranges from 40 to 60°C for optimal extraction of polyphenol compounds. Being the flavonoids within the bioactive compounds, the most stable to the thermal effect, being able to be extracted at temperatures higher than 150°C. Temperature and pressure adjustment guarantees the optimization of the PLE concerning high extraction yields but in conjunction with a reduction of the organic solvent used and shorter process times (Santos et al., 2012; Evstafev and Chechikova, 2016). These characteristics make the PLE as a superior process when compared to other researches that used conventional techniques to project a larger scale of extraction of polyphenols from food waste by-products (Wijngaard et al., 2012; Evstafev and Chechikova, 2016).

##### pH

The effect of the solvent pH, adjusting the temperature or including a buffer, is crucial where target compounds are soluble at selected pH levels. Few studies report the effect of pH in PLE, the works found in literature mention the anthocyanins as the main compound extracted when the pH is altered. Anthocyanins are phenolic compounds belonging to the flavonoid family responsible for the colors of the petals of flowers and fruits; anthocyanins are water-soluble vacuolar pigments that may appear as red, purple, or blue depending on pH values (Bleve et al., 2008; Ren et al., 2020). Pereira et al. (2019) performed PLE for anthocyanins extraction using ethanol-water pH 2.0 (50% w/w) as a solvent. The anthocyanins recovery in acid medium is increased due to their stability in pH from 1.0 to 3.0. The results show that low pH helped to increase their extraction yield.

Espada-Bellido et al. (2018) carried out the extraction of anthocyanins from Mulberry (*Morus nigra* L.) by PLE, investigating different operating conditions: solvent, temperature, pressure, purge time, pH: 3–7, and flushing, the authors obtained an improvement in the yields of anthocyanins as pH decreased from 7 to 3. However, the yields for total phenolic compounds increased with a pH of 7. Therefore, more studies need to be carried out to investigate the recovery of the extraction of flavonoids by altering the pH.

##### Flow rate and extraction time

The flow rate is a parameter in the process of extracting great importance of research, which is directly related to the residence time. Higher flow rates result in shorter residence times of analytes, as well as the rapid extraction of compounds extracted from the plant matrix (Plaza and Turner, 2015; Lachos-Perez et al., 2018). The majority of PLE works use relatively low flow rates (Kamali et al., 2016; Corazza et al., 2018; Pereira et al., 2019) because phenomenologically the desorption and diffusion of the solvent within the pores of the plant matrix used in the extraction process (Plaza and Turner, 2015). Some studies in the literature report flow rates of 0.2, 0.4, 0.6, 0.8, 1 mL/min (Kamali et al., 2016), others report flows of 1.0, 2.0, and 3.0 mL/min for pressurized liquid extraction (PLE) for goldenberry, passion fruit rinds, citrus by-products (Viganó et al., 2016; Barrales et al., 2018; Corazza et al., 2018).

In terms of rapid extraction, the process is more careful, because the thermal effect can form other compounds; the continuous flow of solvent that enters the extraction cells has the objective of extracting the soluble analytes. If the solubility of the compounds in the solvent is limited, the flow velocity must be varied, in the manner of reducing the residence time of the components soluble in water at high temperature, this increase in the flow rate is more effective in terms of using lower amounts of solvent. A study conducted in 2018 reported an extraction time of 60 min for PLE of polyphenols from Goldenberry (Corazza et al., 2018). Another study used a similar extraction time and obtained high yield for PLE of β-glucans and phenolic compounds (Benito-Román et al., 2015). However, it can lead to low solute concentrations within the collected liquid. What originated the use of other operations to reduce the solvent content within the liquid sample to concentrate the extract (Liu et al., 2014).

### Supercritical Fluid Extraction

#### Fundamentals, Main Influencing Parameters, and Applications

Supercritical fluid extraction (SFE) is a green extraction technology that has been widely applied for the recovery of valuable compounds from different materials, both at the laboratory and industrial levels (Herrero et al., 2015). In some cases, laboratory-scale studies were performed to fully recover the compounds of interest for quantification purposes, while in other cases, the aim was to provide optimum conditions for the recovery of bioactive compounds to increase the scale for commercial extraction. The most well-known examples in which scaling has been carried out are decaffeination of coffee beans (Zosel, 1981) and extraction of α-acids from hops to produce hop resins (Laws et al., 1980).

In SFE, the extractor is in its supercritical state, which means that both pressure and temperature are above their critical values. Supercritical fluids have intermediate properties between those of gas and liquids, which depend on the pressure, temperature, and composition of the fluid. In particular, their viscosity and surface tension is similar to that of liquids. At the same time, the diffusion coefficients are similar to those of gases, which allows for more efficient extractions by diffusing more quickly through the solid matrix (Azmir et al., 2013). These properties make it suitable for the extraction of compounds in a short time with higher yields (Azmir et al., 2013). Also, the density (and hence the solvation power of the fluid) can be adjusted by varying the pressure and temperature, providing the opportunity to theoretically perform highly selective extractions (Camel, 2001). Because of the mentioned advantages, the extraction with supercritical fluid is a process of growing interest in areas such as food, pharmaceutical, and cosmetic industries. This method is a powerful tool in the case of the extraction of natural compounds from food products (Justyna et al., 2017).

**Figure 4:**
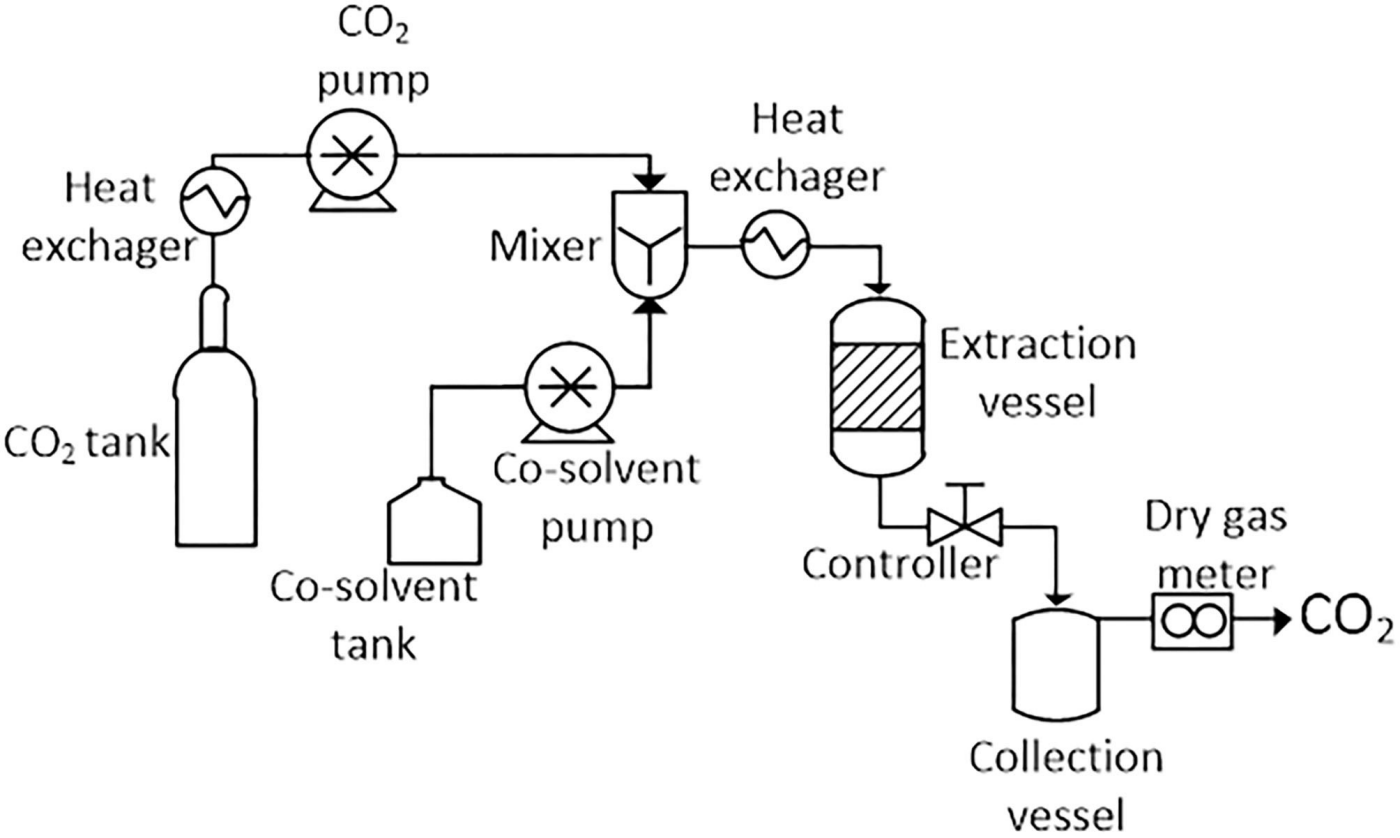
Schematic diagram of a typical supercritical fluid extractor. Adapted of Azmir et al. (2013) and Yahya et al. (2018).

A basic SFE system consists of the following parts: a mobile phase tank (generally CO_2_), a pump for transporting and pressurizing the solvent, cosolvent vessel, and pump, a heater for heating the solvent or the supercritical mixture, a pressure vessel where it occurs the extraction, a controller to maintain the high pressure of the system and a collection vessel to collect the extract. A schematic diagram of the typical SFE unit is shown in Figure 4. Individual units may have different configurations, instrumentation, valves, by-pass, gas purging systems, and safety features not shown here (Azmir et al., 2013; Yahya et al., 2018).

SFE can be performed in static or dynamic mode. For processing, the raw material is placed in the extractor vessel, in which the temperature and pressure are controlled. The extractor vessel is then pressurized with fluid by the pump. Afterward, the analyte is collected in the collection vial due to solvent depressurizing. The fluid is then rejuvenated and recycled or released into the environment in the last step (Camel, 2001; Yahya et al., 2018).

Although a variety of solvents may be employed in supercritical conditions, such as those mentioned in Table 5, carbon dioxide (CO_2_) is by far the most widely used supercritical fluid for the recovery of bioactive compounds from natural matrices (Silva et al., 2016). CO_2_ has several advantages, including moderate critical conditions (Tc ≈ 31 °C and Pc ≈ 74 bar), inert, non-toxic, non-flammable, non-explosive, environmentally friendly, recognized as safe by the European Food Safety Authority (EFSA) and by the Food and Drug Administration (FDA) of the United States of America. Furthermore, it is cheap and readily available (Herrero et al., 2015; Yahya et al., 2018). Another advantage is that CO_2_ is gaseous at ambient temperature and pressure, which makes the recovery of the analyte solvent-free and straightforward. These characteristics are of significant interest for the production of bioactive compounds to be used in the food, pharmaceutical, and cosmetic industry. It avoids subsequent steps to remove the extractor solvent, which are usually needed when other modern extraction techniques are chosen to capture bioactive from natural sources. In addition, SFE using CO_2_ allows the extraction of easily oxidizable or thermosensitive compounds (which are characteristic of some flavonoids) by operating at low temperatures using a non-oxidizing medium (Herrero et al., 2010). The only drawback of CO_2_ is its low polarity, which makes it ideal for lipid, greasy, and non-polar substances (such as carotenoids, aromas, volatile compounds), but unsuitable for polar flavonoids.

**Table 5:** Critical properties of some pure components used in SFE (Sahena et al., 2009; Silva et al., 2016).

**Component**	**Critical property**		
	**Temperature (°C)**	**Pressure (MPa)**	**Density (g/cm^3^)**
Ethylene (C_2_H_4_)	9.4	5.04	0.215
Carbon dioxide (CO_2_)	31.1	7.38	0.470
Ethane (C_2_H_6_)	32.4	4.82	0.203
Nitrous oxide (N_2_O)	36.7	7.17	0.460
Sulfur hexafluoride	45.8	3.77	0.730
Propylene (C_3_H_6_)	91.9	4.60	0.232
Propane (C_3_H_8_)	96.8	4.25	0.217
Freon-134a (CH_2_FCF_3_)	101.1	4.06	0.512
Freon-12 (CCl_2_F_2_)	111.9	4.14	0.565
Acetone (C_3_H_6_O)	235.1	4.70	0.278
Methanol (CH_3_OH)	239.6	8.09	0.272
Ethanol (C_5_H_5_OH)	240.9	6.14	0.276
Ethyl acetate (C_4_H_8_O_2_)	250.2	3.83	0.360
Water (H_2_O)	374.1	22.06	0.322

The limitation of the low polarity of CO_2_ has been successfully overcome by the use of chemical modifiers or cosolvents, such as methanol, water, acetone, ethanol, and acetonitrile. These cosolvents can increase the polarity of the supercritical fluid mixture, and consequently, the solvation concerning the target bioactive compounds (Azmir et al., 2013; Bubalo et al., 2018; Yahya et al., 2018). These are employed during extraction in small proportions, typically 1–10% (Herrero et al., 2015). Modifiers may be introduced as mixed fluids into the pumping system with a second pump and a mixing chamber (see Figure 3), or by merely injecting the modifier as a liquid in the sample before extraction. The second way is less successful because it leads to the formation of concentration gradients within the sample. The third very rarely applied form is the use of a cylinder tank with modified CO_2_ (Bubalo et al., 2018).

Among the cosolvents for SFE, ethanol is considered the best for the extraction and fractionation of bioactive compounds, such as flavonoids, for nutraceutical and food application, due to its lower toxicity and less selectivity when compared to methanol and other organic solvents. Inversely, water is not generally used as the only cosolvent for SFE, primarily because of its low solubility in CO_2_ (Bubalo et al., 2018). Therefore, binary mixtures of water and ethanol are generally employed for extracting SFE (Yahya et al., 2018). The properties of the sample and the solutes of interest, the affinity of the application, and the previous results published in the literature are the primary basis for the selection of the best modifier (Azmir et al., 2013). Table 6 summarizes the most relevant published works in the last 3 years using the SFE technique, using pure supercritical CO_2_ and CO_2_ mixed with a cosolvent (water and/or ethanol) for the extraction of flavonoids.

**Table 6:** Some representative applications involving the use of SFE for the recovery of flavonoids from natural sources.

**Natural source of flavonoids**	**Solvent**	**Co-solvent**	**Extraction mode**	**T (°C)/P (MPa)**	**Flow rate**	**Time (mim)**	**Target compounds**	**Yield**	**Reference**
Black poplar buds	CO_2_	–	Dynamic	40-60/12-30	2 kg/h	60	Individual flavonoids	0.42–79.56 μg/mg	Jakovljevi et al., 2018
Haskap berry pulp	CO_2_	Water (2.2–5.5 g)	Static + dynamic	35-65/10-52	10 mL/min	15–120	Anthocyanins	16.6–52.7%	Jiao and Kermanshahi, 2018
Brown onion skin	CO_2_	Ethanol 85% (5–15%)	Dynamic	10/40	10 mL/min	120	Quercetin and derivatives	65–118%	Campone et al., 2018
*Odontonema strictum* leaves	CO_2_	Ethanol 95% (15%)	Static + dynamic	55-65/20-25	15 g/min	210–270	Total flavonoids	10.68–18.92 mg/g	Ouédraogo et al., 2018
Cipó-pucá	CO_2_	Ethanol (0–10%)	Static + dynamic	40-60/20-40	4.52 g/min	210	Total flavonoids	0.28–13.44 mg/g	Salazar et al., 2018
*Opuntia ficus indica* (L.) Mill	CO_2_	Ethanol (5%)	Dynamic	35-65/20-40	60–100 g/min	120	Isorhamnetin conjugates	12.45–188.07 mg/100 g	Antunes-Ricardo et al., 2017
*Citrus unshiu* peels	CO_2_	Ethanol (0–50 mol%)	Dynamic	60/30	0.026 mol/min	180	Nobiletin	0.13–0.57 μg/g	Oba et al., 2017
Tea leaves	CO_2_	Ethanol (1–3 g/min)	Static + dynamic	40-60/10-20	8 g/min	60	Total flavonoids	1.87–1.95 mg/mL	Maran et al., 2015
Myrtle leaves	CO_2_	Ethanol	Dynamic	45/23	0.3 Kg/h	120	Quercetin and myricetin	2–8 (10^−3^) mg/g	Pereira et al., 2016
*Hibiscus sabdariffa*	CO_2_	Ethanol (7–15%)	Dynamic	40-60/15-35	25 g/min	90	Methyl-epigallocatechin	0.34–0.53 mg/g	Pimentel-Moral et al., 2018a
Agroindustrial soybean residue	CO_2_	Ethanol (3%)	Dynamic	35-40/40	0.5 Kg/h	–	Total flavonoids	31.3–65.0 mg/100g	Alvarez et al., 2019
Radish leaves	CO_2_	Ethanol	Dynamic	35-50/30-40	0.6 Kg/h	–	Total flavonoids	13.9–21.8 mg/100g	Goyeneche et al., 2018

It is noted in some of these studies that the use of cosolvent significantly improved the yield of the target flavonoid extraction. For example, (Salazar et al., 2018), achieved higher yields (9.01–13.44 mg QE/g of extract) of total flavonoids employing 10% cosolvent when compared to the conditions in which was used pure CO_2_ (0.14–1.08 mg QE/g of extract) and supercritical mixtures with lower cosolvent content (3.70–5.52 mg QE/g of extract). Behavior similar to that was verified by Oba et al. (2017) in the capture of flavonoid nobiletin. Also, the use of modifiers/cosolvents in the SFE process produces extracts with flavonoid yields as good as those produced by other emerging extraction techniques mentioned earlier in this work. Garcia-Mendoza et al. (2017) when extracting anthocyanins from juçara (*Euterpe edulis* Mart.) residue obtained higher extract yields (22.0 mg/g dry residue) using supercritical extraction with CO2-water-ethanol (90 CO_2_ and 10% cosolvent) compared to the acidified hydroethanolic extract obtained by the PLE technique (7.7 mg /g dry residue). Similarly, Song et al. (2019) noted an improvement in the recovery of kaempferol and quercetin glycosides from Xinjiang jujube (*Ziziphus jujuba* Mill.) leaves, when employing the SFE-CO_2_/ethanol technique compared to UAE extraction.

In addition to the supercritical solvent, modifier nature, modifier proportions, other factors such as physical-chemical characteristics of the vegetable matrix (moisture content, particle size, chemical composition, etc.), solvent flow rate, extraction mode, extraction time, solvent-feed ratio, the porosity of the extraction bed, diameter-extractor length ratio, extractor free volume, and highlighted, pressure and temperature are involved in the extraction process of flavonoids by SFE (Pourmortazavi and Hajimirsadeghi, 2007). In previous papers (Pourmortazavi and Hajimirsadeghi, 2007; Bubalo et al., 2018), there is a detailed review of the influence of these factors/parameters on the SFE system.

In general, the extraction efficiency of bioactive compounds is increased by increasing the pressure and temperature of the supercritical CO_2_, to an ideal level (Molino et al., 2020). It happens, for the reason that both parameters play a significant role in the solubility of solutes in the solvent (Bubalo et al., 2018; Molino et al., 2020). At constant temperature, the increase in pressure causes an increase in the density of CO_2_ and its solvation power, which consequently improves the diffusivity and mass transfer of the target compounds into the fluid (Silva et al., 2016; Molino et al., 2020). At constant pressure, by increasing the temperature of the medium, the fluid becomes less viscous, and the vapor pressure of the solutes increases, leading to a rise in the extraction yield (Bimakr et al., 2011; Bubalo et al., 2018). However, there are studies that, for specific temperature ranges employed, have noticed an opposite effect on this. Wang et al. (2008) noted that total flavonoid yield, extracted from the Chinese medicinal plant *Pueraria lobata*, decreased from 16.8 mg/g to 14.2 mg/g when the temperature was changed in the range of 50–60°C. According to reports contained in this work and the specific literature, this happens because a moderate increase in temperature, when applied at relatively low pressure, can lead to a sharp decrease in the density of CO_2_ with a consequent reduction in the solubility of the target compounds (Wang et al., 2008; Essien et al., 2020; Molino et al., 2020). A similar effect was observed in the studies of Liu et al. (2011) and Song et al. (2019), which extracted flavonoids from the Asian species *Maydis stigma* and *Ziziphus jujuba* Mill., respectively.

In addition to the ideal temperature and pressure conditions, maximizing the contact of the supercritical solvent with the flavonoid source plant material is necessary to obtain the best efficiency of the SFE extraction. Extraction time, solvent flow rate, and extraction mode (static and dynamic) are variables that directly influence the contact of the solvent with the sample material (Pourmortazavi and Hajimirsadeghi, 2007). The introduction of a static period before dynamic extraction is used to allow the solvent to penetrate the plant matrix and, therefore, improve the mass transfer of the solute to the fluid by diffusivity, besides increasing the yield, and reducing the total extraction time (Alexandre et al., 2020). Static periods between 10 and 30 min are the ones that have been most used to capture phenolic compounds of the flavonoid class (Wang et al., 2008; Liza et al., 2010; Song et al., 2019; Yang et al., 2019). For dynamic extraction, a more extensive range is observed, reaching periods of up to 270 min (Ouédraogo et al., 2018; Yang et al., 2019). However, it is essential to indicate that extraction time is determined by the quantity and composition of the feed raw material. Generally, the extraction yield increases with the increase in the extraction time until the solubility reaches its limit. (Song et al., 2019) found a significant increase in total flavonoids when the time changed from 30 to 120 min. Ouédraogo et al. (2018) observed similar behavior when extracting polyphenols from *Odontonema strictum* leaves. However, in the study by Jiao and Kermanshahi (2018), no significant effect was noted when extracting anthocyanins from haskap berry pulp in periods 15–120 min of static time and 20–60 min of dynamic time.

The CO_2_ solvent flow rate or the supercritical mixture (CO_2_-modifier) directly influences the contact time and the mass transfer phenomenon by convection. Typically, an increase in the solvent flow rate increases the extraction capacity by reducing the external resistance to mass transfer and increasing the chances of intermolecular interactions between the solvent and the solutes (Essien et al., 2020; Molino et al., 2020). Ruslan et al. (2018) recovered a higher catechin content (565.38 ppm) from betel nuts at the highest flow rate studied (4 mL/min). However, as noted in a previous study (Ruslan et al., 2015), very high solvent flow rates can substantially reduce extraction yield due to insufficient contact time between the compounds and the extraction fluid (Essien et al., 2020).

As can be seen, in general, all parameters of the SFE process can be adjusted and, depending on the extraction conditions, can interact with each other to maximize the extraction efficiency. For this reason, experimental designs to determine the best extraction conditions in a particular SFE extraction process are widely needed and used so that a systematic study can be performed with a statistically supported selection of influential variables (Camel, 2001; Azmir et al., 2013; Herrero et al., 2015). The knowledge of these effects is also useful for the economic evaluation of the process to predict the capacity of the process in an increase of scale and to optimize an industrial plant (Bubalo et al., 2018).

## Conclusions

There is a clear and growing interest in the extraction and isolation of natural products and their applications. The primary importance of modern analytical research is innovative and safe extraction processes, which are economically viable, and environmentally correct for higher recovery of bioactive compounds in shorter process time from natural sources. The advantages of such techniques have been immense in the production of extracts with good quality and yield, lower consumption of solvent, energy, and shorter extraction time. The most appropriate extraction technique depends on plant matrices and the type of compost, and defined selection criteria should be followed. Recent studies have shown that green extraction methods offer excellent alternatives to traditional methods. Many studies are still being carried out in this field, to improve these new green extraction techniques further, with the intention always to reduce the cost of extraction, the time consumed, the quality of the extract, the environmental safety, and health. Also, the combination of extraction methods usually presents advantages to overcome the limitations of a particular approach. Ironically, the massive volume of information available regarding the extraction of flavonoids from the most diverse types of samples makes it difficult to draw overall conclusions. However, the variability of the results reported in the literature for similar compounds is intrinsically related to the sample characteristics, which play a critical role in releasing them to the extraction 6 solvent. Fortunately, our knowledge about the process and how the variables can be used to control the process is increasing at a fast pace, which is leading to innovative approaches to maximize yields and reduce degradation while at the same time minimizing its environmental impact.

## Author Contributions

All authors made a significant contribution to this article. All were involved in in the idea and in the writting of specific parts. JC, MS, LS, and MR were responsible for the introduction, ultrasound-assisted extraction, and reviewing whole the manuscript. DL-P, PT-M, and TF-C were responsible for the section of pressurized liquid extraction. MV-E, AG-d-P, and GB were responsible for the microwave-assisted extraction part. AM was responsible for the supercritical fluid extraction part of the manuscript. All authors were involved in the conpetual criation and reviewing the manuscript.

## Conflict of Interest

The authors declare that the research was conducted in the absence of any commercial or financial relationships that could be construed as a potential conflict of interest.
